# The evolution of machining-induced surface of single-crystal FCC copper via nanoindentation

**DOI:** 10.1186/1556-276X-8-211

**Published:** 2013-05-04

**Authors:** Lin Zhang, Hu Huang, Hongwei Zhao, Zhichao Ma, Yihan Yang, Xiaoli Hu

**Affiliations:** 1 College of Mechanical Science & Engineering, Jilin University, Renmin Street 5988, Changchun, Jilin 130025, China

**Keywords:** Machining-induced surface, Nanoindentation, Nucleation of dislocations, Molecular dynamics simulation

## Abstract

The physical properties of the machining-induced new surface depend on the performance of the initial defect surface and deformed layer in the subsurface of the bulk material. In this paper, three-dimensional molecular dynamics simulations of nanoindentation are preformed on the single-point diamond turning surface of single-crystal copper comparing with that of pristine single-crystal face-centered cubic copper. The simulation results indicate that the nucleation of dislocations in the nanoindentation test on the machining-induced surface and pristine single-crystal copper is different. The dislocation embryos are gradually developed from the sites of homogeneous random nucleation around the indenter in the pristine single-crystal specimen, while the dislocation embryos derived from the vacancy-related defects are distributed in the damage layer of the subsurface beneath the machining-induced surface. The results show that the hardness of the machining-induced surface is softer than that of pristine single-crystal copper. Then, the nanocutting simulations are performed along different crystal orientations on the same crystal surface. It is shown that the crystal orientation directly influences the dislocation formation and distribution of the machining-induced surface. The crystal orientation of nanocutting is further verified to affect both residual defect generations and their propagation directions which are important in assessing the change of mechanical properties, such as hardness and Young's modulus, after nanocutting process.

## Background

Ultraprecision machining at nanometric scale is increasingly required in micromachining and nanomachining to produce parts of intricate features and surface finish quality [1]. Material removal at such a small uncut chip thickness involves subsurface deformation, and in conventional cutting, the effect of subsurface deformation is neglected as the uncut chip thickness is significant. However, it is not the same case in nanocutting due to the small uncut chip thickness on the order of several nanometers or less [2]. Thus, the effect of subsurface deformation should not be neglected as the uncut chip thickness is in the same scale. Subsurface deformed layer is related to the deformation and damage in the material especially in the micro- and nanoscales, in which not only the size is reduced substantially but also the physical characteristics on optics and electricity of the material become different. Recently, the mechanisms of subsurface deformation have become the key issues to be investigated.

Many investigations have been conducted to study the subsurface deformed layers during nanocutting process via molecular dynamics (MD) simulations. Shimada and Ikawa et al. performed MD simulation of microcutting of free machining materials under perfect motion of a machine tool. Based on the radial distribution function, they found that the ultimate depth of the deformed layer of a specimen is 5.0 nm [3-5]. Zhang et al. conducted MD simulation of nanometric cutting on single-crystal copper. A new criterion based on single-atom potential energy variation was established [2]. However, the previous studies evaluated the subsurface atom deformation behaviors mainly by studying and analyzing the cutting forces and potential energy variations. Although these features of different deformation behaviors can be revealed efficiently, the potential energy variation of atoms is hardly measured by current experimental equipment. Therefore, it is an important issue to investigate the surface properties of the subsurface deformed layers after nanocutting process.

Nanoindentation is the most frequently used technique to measure surface properties such as Young's modulus and hardness [6]. Investigations on exploring the performances of friction and wear of single-crystal materials are thus of scientific and technological interest. For this reason, a lot of studies on nanoindentation based on experimental and various theoretical models have been carried out to have deep understanding of the performance of these surface and near-surface tribological properties. Yan et al. performed nanoindentation tests on ultraprecision diamond-turned silicon wafers [7,8]. Compared with those of pristine silicon wafers, the machining-induced amorphous layer was with significantly higher microplasticity and lower hardness than pristine silicon. Zhao et al. performed the same process and analyzed the machinability of the material and its structure via molecular dynamics simulation [9]. Although the experimental and theoretical results revealed the structure transformation in diamond semiconductors, the mechanism of the phase transformation did not suit for most of metal materials. Since the lattice structure of a metal is different from a semiconductor, the phase transformation is not fitful for most face-centered cubic (FCC) metals. Consequently, understanding of the different performances and machinability of the machining-induced layer in a FCC metal becomes essential.

In this paper, theoretical analysis and investigation on the properties of subsurface deformed layers in nanocutting process with the aid of nanoindentation test will provide much information on the mechanisms of the deformation in the material. The displacements of dislocations are simulated to have better understanding of the mechanism of the damaged layer in nanocutting and nanoindentation test on a machining-induced surface. The remainder of this paper is organized as follows: The ‘Methods’ section gives the models and conditions of the MD simulation. The ‘Results’ section presents the results of the simulation and discusses the results in detail. The ‘Discussion’ section discusses the effect of cutting directions along different crystal orientations on the subsurface deformed layers. The last part draws some interesting conclusions.

## Methods

### Simulation model

**Figure 1:**
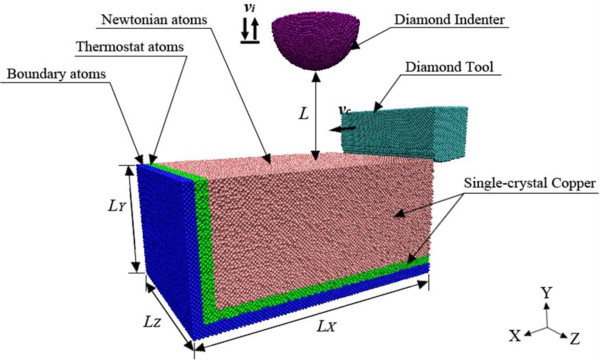
**Schematic diagram of three-dimensional MD model of single-crystal copper for nanoindentation with hemispherical indenter after nanocutting.** The size of the control volume is *L*_*X*_ × *L*_*Y*_ × *L*_*Z*_ = 27.112 nm × 12.65 nm × 18.07 nm. In all the calculations, the velocity of the diamond tool *v*_*c*_ = 200 ms^−1^ and the velocity of the indenter *v*_*i*_ = 30 ms^−1^.

A schematic diagram of the three-dimensional MD simulation model is shown in Figure 1. The model consists of a single-crystal copper specimen, a diamond tool, and a hemispherical diamond indenter. The specimen size is 75*a* × 35*a* × 50*a* along the *X*, *Y*, and *Z* directions, consisting of 525,000 atoms, where *a* is the lattice constant of Cu (0.3614 nm). The copper atoms in the specimen are categorized into three kinds of atoms: boundary atoms, thermostat atoms, and Newtonian atoms. The boundary atoms are fixed in space to reduce the boundary effects and maintain the proper symmetry of the lattice. The motion of Newtonian atoms is determined by the force restricted by Newton's equation of motion. The thermostat atoms are used to ensure reasonable outward heat conduction away from the machined zone.

The diamond tool consists of 21,823 carbon atoms, and the rake angle and clearance angle are 0° and 7°, respectively. For conventional cutting processes, the cutting tool edge is usually assumed to be very sharp, that is, the edge radius is negligible. However, during nanocutting process of materials, this assumption is not reasonable since the cutting tool edge radius is on the same scale as the undeformed chip thickness. Thus, the simulation has been done with the cutting edge radius of 2 nm. The spherical indenter contained 36,259 atoms with a radius of 50.0 Å.

The motions of the atoms in the Newton and thermostat atoms are assumed to follow Newton's law of motion which can be computed from the interatomic forces as follows:

(1) $$a_{\mathrm{ix}}=\frac{F_{\mathrm{ix}}}{m_{i}}=\frac{d^{2}x_{i}}{dt^{2}}\;F_{\mathrm{ix}}=-\frac{\mathrm{dV}}{dx_{i}}$$

where *a*_*ix*_ represents the *i* atom's acceleration in the *X* direction, *m*_*i*_ is the mass of the *i* atom, *F*_*ix*_ is the interaction force between the *i* atom by the *j* atom in the *X* direction, *x*_*i*_ indicates the *i* atom's *X*-coordinate, and *V* is the potential energy.

The temperature of atoms during the machining simulation can be calculated using the conversion between the kinetic energy and temperature as follows:

(2) $$\frac{1}{2}\sum_{i}m_{i}v_{i}^{2}=\frac{3}{2}Nk_{b}T$$

where *N* is the number of atoms in groups, *v*_*i*_ represents the velocity of the *i* atom, *k*_*b*_ is the Boltzmann constant which is equal to 1.3806503 × 10^−23^ J/K, and *T* represents the temperature on atoms.

In order to keep the temperature constant during the nanocutting process and nanoindentation process, in other words, ensuring reasonable heat conduction outwards from the Newtonian atom zone [10], the thermostat atom zone is set to absorb the heat from the specimen. When the temperature of the thermostat atom zone is higher than the preset one of 296 K, the velocity rescaling method as shown in Equation 3 [11] is used to control the temperature of the thermostat atom zone and absorb the heat towards the Newtonian atom zone. The direct velocity scaling method was employed to maintain the total kinetic energy at a constant value. The velocity of every atom in the thermostat atom zone needed to be scaled at every integrating step, and the velocity scaling factor is as follows:

(3) $$\beta ={\left[3Nk_{b}T/\sum_{i}m_{i}v_{i}^{2}\right]}^{1/2}$$

### Selection of potential energy function

In this paper, there are two kinds of atoms in the MD simulation model, which are C and Cu atoms. Therefore, there are three different atomic interactions between them, which are the interaction between single-crystal copper atoms (Cu-Cu), the interaction between diamond atoms (C-C), and the interaction between copper atoms and diamond atoms (Cu-C) or (C-Cu).

The potential energy function affects the accuracy of the simulation which governs the reliability of results. Between copper atoms in the specimen, the embedded atom method (EAM) potential [12] was applied to describe the Cu-Cu interaction. The EAM potential, which evolved from the density function theory, is based on the recognition that the cohesive energy of a metal is governed not only by the pair-wise potential of the nearest neighbor atoms, but also by embedding energy related to the ‘electron sea’ in which the atoms are embedded. For the EAM potential [12], the total potential energy of the system is expressed as follows:

(4) $$E_{\mathrm{tot}}=\frac{1}{2}\sum_{\mathrm{ij}}\phi_{\mathrm{ij}}\left(r_{\mathrm{ij}}\right)+\sum_{i}F_{i}\left(\rho_{i}\right)$$

where *E*_tot_ is the total potential energy of the system, *φ*_*ij*_ is the pair potential between atoms *i* and *j*, *r*_*ij*_ is the distance between the atoms *i* and *j*, and *F*_*i*_(*ρ*_*i*_) is the embedded energy of atom *i*. *ρ*_*i*_ is the host electron density at atom *i* induced by all of the other atoms in the system as follows:

(5) $$\rho_{i}=\sum_{j\neq i}\rho_{j}\left(r_{\mathrm{ij}}\right)$$

where *ρ*_*i*_ (*r*_*ij*_) is the contribution to the electronic density at the site of the atom *i*, and *r*_*ij*_ is the distance between the atoms *i* and *j*.

Because diamond is much harder than copper, the diamond tool and indenter are both treated as a rigid body in the simulation. Therefore, the atoms in the tool are fixed to each other relatively, and no potential is needed to describe the interaction between diamond atoms (C-C) [13].

The interaction between copper atoms and diamond atoms (Cu-C) is described by the Morse potential [14]. Although a two-body potential may lead to less accurate solutions than a many-body potential does, its parameters can be accurately calibrated by spectrum data. For the Morse potential [14], the two-body potential energy is expressed as follows:

(6) $$V\left(r_{\mathrm{ij}}\right)=D\cdot \left(e^{-2\alpha \left(r_{\mathrm{ij}}-r_{0}\right)}-2e^{-\alpha \left(r_{\mathrm{ij}}-r_{0}\right)}\right)$$

where *V*(*r*) is the potential energy, *D* is the cohesion energy, *α* is the elastic modulus, and *f*_*ii*_ is the second derivative of the potential energy *V*(*r*) with respect to the bond length *r*_*ij*_. *r*_*ij*_ and *r*_*0*_ are the instantaneous and equilibrium distances between two atoms, respectively. Table 1 shows magnitudes of these parameters.

**Table 1:** **Parameters in the standard Morse potential**[14]

**C-Si**	**Parameter**
*D* (eV)	0.087
*α* (Å^−1^)	5.14
*r*_*0*_ (Å)	2.05

### MD simulation setup

In order to reduce the boundary effect and size effect, the model scale should be large. As a result, the simulation becomes computationally expensive. To avoid these problems, the periodic boundary condition is set along the *Z* direction [14]. The specimen surface of the *X-Z* plan is machined, so it is a free surface. Both the diamond tool and the diamond indenter are set as a rigid body. This was followed by an energy minimization to avoid overlaps in the positions of the atoms. The simulation model was equilibrated to 296 K under the microcanonical (NVE) ensemble, and the initial velocities of the atoms were assigned in accordance with the Maxwell-Boltzmann distribution.

**Figure 2:**
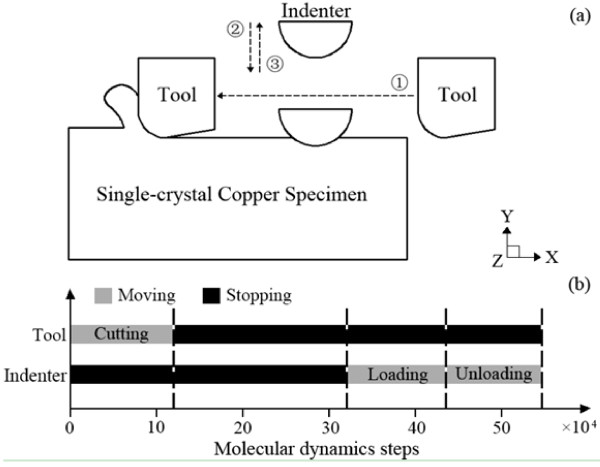
**Schematic of nanoindentation tests on machining-induced surface and traces of the diamond indenter and diamond tool.** (**a**) The motivation sequence of the diamond tool and indenter in the simulation and (**b**) the time series of the simulation.

Figure 2 shows the simulation procedure of the nanoindentation test on the machining-induced surface. Firstly, the diamond tool cuts the surface along the [ī00] direction for the first time in the *X-Z* plane (Figure 2a, (1)). After the nanocutting stage, the relaxation starts, in which the tool is fixed in its final position and the fixed boundaries are removed so that the system can be relaxed back to another state of equilibrium (Figure 2b). Then, the diamond indenter moves along the [00ī] direction (as shown in Figure 2a (2) and returns to its initial position (3)).

The nanocutting proceeds along the [ī00] direction in the (010) surface. In the simulation, the cutting speed is set at 200 m/s. Since the rates of cutting speed, loading, and unloading of the MD simulations are much higher than those of the experiments, only a qualitative prediction of the structural transformation is obtainable [2]. More parameters used in the current simulation model are listed in Table 2.

**Table 2:** Computational parameters used in the MD simulation model

	**Material**		
	**Substrate: copper**	**Tool: diamond (rigid)**	**Indenter: diamond (rigid)**
Potential function	EAM potential function	None	None
Dimensions	75*a* × 35*a* × 50*a*	Rake angle, 0°	Hemisphere indenter
	(*a* is the lattice constant, 0.3614 nm)	Clearance angle, 7°	Radius, 50.0 Å
Time step	0.1 fs		
Original temperature	296 K		
Number of atoms	525,000	21,823	36,259
Cutting depth	1.0 nm		
Cutting velocity	[ī00] on (010) surface	200 m/s	
Indentation depth	2.0 nm		
Indentation velocity	[010] on (010) surface		50 m/s

The three-dimensional MD simulations were performed by the large-scale atomic/molecular massively parallel simulator (LAMMPS)^a^ developed by Plimpton et al. [11,15]. The parallel computation was realized under the help of message passing interface library.

## Results

### Description of interior defects in nanocutting

**Figure 3:**
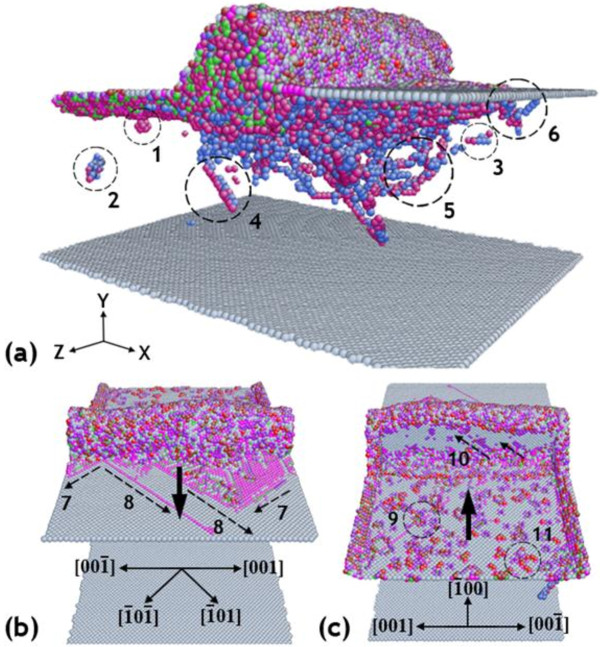
**Dislocations distributed in the specimen at the instant of 16.8-nm nanocutting distance.** (**a**) The interior defects inside the specimen. (**b**) The front view on the machining surface. (**c**) The rear view of the machining surface.

Before investigating the machining-induced surface mechanical properties by nanoindentation, we present in this section a general description of the phenomenon observed on and beneath the machining-induced surface Cu (010) in the simulations of nanocutting process. Figure 3 shows the views at the instant of 16.80-nm nanocutting distance with three different perspective angles. The cutting direction is along the [ī00] direction, and the penetration depth is set at 1.0 nm, with 200 ms^−1^ cutting velocity on the Cu (010) surface. The color in Figure 3 represents the atomic coordinated numbers of the copper atoms in the specimen. The atoms with a coordination number of 12 that depict copper atoms have been deliberately eliminated in the visualization so that we can clearly see any changes to the crystalline order of single-crystal FCC copper. The rest of the atoms and structures in Figure 3 only involve boundary atoms and defect-related atoms.

According to Figure 3a, there are several different defects generated during the nanocutting process. Various defects distributed in the specimen are marked by the numbers in Figure 3a. The single vacancy, marked with number 1, is easily identified by its simple dependent structure and atomic coordinated number. When the dependent single vacancies are gathered by the movements and interactions of dislocations in the specimen, the immobile vacancy clusters and a vacancy chain (marked with 2 and 3 in Figure 3a) are generated beneath the machining-induced surface, which may largely alter the mechanical properties of the machined surface after nanocutting.

The threading dislocation, marked with number 4, belongs to one of the mobile defects in the specimen. It is well shown that the threading dislocation marked in the specimen is parallel with the slip vectors associated with the FCC (111) surface. According to the position-sensitive criterion [16], its motion in the specimen under the machining-induced surface determines the plastic deformation of the material in nanocutting.

The dislocation loop of numbers 5 and 6, which was emitted from the tool-specimen interface, denotes the dislocation loops. Unlike the single vacancy defects distributed in the specimen, the dislocation loops glide along with the movement of the diamond tool. In addition, the motion directions of the dislocation loops are not the same. Some dislocations penetrate into the specimen towards the bottom surface, while others are moving along the cutting direction beneath the machining surface. Their motivation promotes not only the nucleation of other defects in the specimen but also theirselves [17]. They initially generated from one side of the specimen and finally went inside the opposite site of the boundary.

Figure 3c provides some different views of the new generated surface. Some dislocation can be seen on the surface. It is also seen that the dislocations on the machining surface marked with numbers 7 and 8 are parallel with the slip vectors [ī0ī] and [ī01]. The two directions in the specimen are the easiest glide vectors in the surface. Many generated dislocations are involved in the accumulated atom pile-up in front of the diamond tool. The black arrow in the figures indicates the cutting direction. Some defects remained on the machining-induced surface, marked with numbers 9 and 11 in Figure 3c. The vacancy-related defects on the machining-induced surface, number 9, are not only immobile but are also located limited on the surface, while the dislocation-related defects are completely contrary. The dislocation loop is usually distributed along with such a defect on the surface. The dislocation nucleation and escape in submicrometer single-crystal FCC metal materials have been observed and proven in some previous studies using experiments and simulations [18,19].

### The nanoindentation test on the machining-induced surface

#### The energy distribution of the machining-induced surface

**Figure 4:**
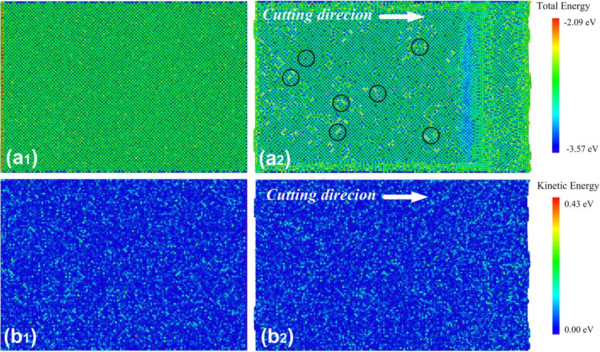
**Atomic total energy distribution and kinetic energy distribution of relaxed machining-induced surface and initial surface.** (**a**_**1**_) and (**a**_**2**_) are the atomic total energy distributions. (**b**_**1**_) and (**b**_**2**_) are the atomic kinetic energy distributions.

The surface physical properties, such as hardness and Young's modulus, of the materials are influenced by many factors, including the initial energy in the material, the initial temperature of the surface, and so on, especially in the testing areas. As we know, if the machining-induced surface exists with a high energy, smaller external energy will be imposed to realize the plastic and elastic deformation, resulting in smaller hardness of the surface. Since the average kinetic energy can be converted into temperature distribution, the kinetic energy distribution is used to present the initial thermal condition. The atomic total energy distribution and kinetic energy distribution of the relaxed machining-induced surface and the initial surface are shown in Figure 4.

Figure 4 (a_1_ and b_1_) shows the atomic total energy distribution and kinetic energy distribution of the initial surface, and Figure 4 (a_2_ and b_2_) shows those of the relaxed machining-induced surface. According to Figure 4, there is no obvious difference in energy distribution on both the relaxed machining-induced surface and the initial surface. Although more high-energy defects are observed to be distributed on the relaxed machining-induced surface (marked with black circles), the overall surface condition is about the same with the initial surface. The result implies that the relax stage after the nanocutting process is well performed for the atomic total energy distribution and that kinetic energy on the surface returns to a low and stable situation. Since the atomic total energy and kinetic energy are about the same as those of the former initial surface, the influential factors due to different energy distributions are well excluded.

#### The interior defects in the nanoindentation tests on the machining-induced surface

The evolution of interior defects inside the specimen during nanoindentation governs the mechanical properties of the surface, especially the hardness and Young's modulus. Therefore, the investigation of the nucleation and penetration of dislocations beneath the indenter seems strongly necessary.

**Figure 5:**
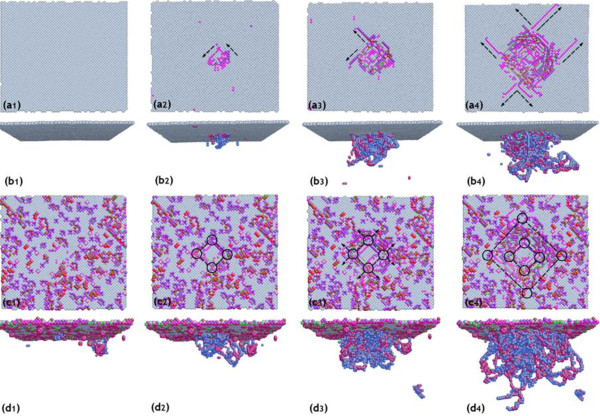
**Sequence of instantaneous defect evolution versus indentation penetration depth.** The sequence of instantaneous defect evolution from the nucleation of dislocation into the formation of dislocation embryos versus indentation penetration depth with top view and front view. (**a**_**1**_) and (**b**_**1**_), 0 nm; (**a**_**2**_) and (**b**_**2**_), 0.5 nm; (**a**_**3**_) and (**b**_**3**_), 1.0 nm; (**a**_**4**_) and (**b**_**4**_), 1.5 nm, respectively. (**c**_**1**_) to (**c**_**4**_) and (**d**_**1**_) to (**d**_**4**_) present a universal process of the dislocation evolution.

In order to evaluate the influence of machining-induced subsurface damages on the mechanical properties of single-crystal copper, a nanoindentation on the pristine single-crystal copper specimen is conducted with the same simulation conditions as the former simulation. Figure 5 shows the sequence of instantaneous defect evolution from the nucleation of dislocation into the formation of dislocation embryos. The evolution of dislocations in the specimen is not the same in the two models.

Figure 5 (a_1_ to a_4_, and b_1_ to b_4_) shows a typical material deformation process of pristine single-crystal copper during nanoindentation with a series of structure evolutions for nucleation of initial defect beneath the indenter. In the undeformed state, none of defects are distributed or generated beneath the indenter. With small deformation, a few vacancies generate just beneath the indenter, which marks the beginning of nucleation of dislocations. As the single-crystal copper atoms experience the displacive structure transition, the well-known dislocation embryos are gradually developed from the sites of homogeneous nucleation as shown in the prospective close-up view of Figure 5 (b_4_). In addition, the atomic glides on the surface are also clearly marked with black arrows, which are parallel with the slip vectors associated with the FCC (111) surface. The motivation of these glides indicates the displacive plastic deformation around the indenter as shown in Figure 5 (b_4_).

Showing contacts to the nucleation of dislocations in the pristine single-crystal copper, the process in the subsurface of the machining-induced surface is different. Figure 5 (c_1_ to c_4_, and d_1_ to d_4_) presents a universal process of the dislocation evolution in the subsurface with initial imperfection of the machining-induced surface. Before the indenter penetrates into the machining-induced surface, there have been some vacancy-related defects distributed on the surface as shown in Figure 5 (c_1_ and d_1_). When the indenter penetrates into the surface, the dislocation embryos are immediately developed from the vacancies around the indenter. Although the glide directions of such defects are still along slip vectors associated with the FCC (111) surface, the initial vacancy-related defects distributed on the machining-induced surface become the beginnings of mobile dislocation loops. The formation energy of mobile dislocation of such a process is largely reduced. In addition, much more dislocation loops in the specimen are motivated by the indenter-specimen interaction, leading to the permanent plastic deformation of the material.

**Figure 6:**
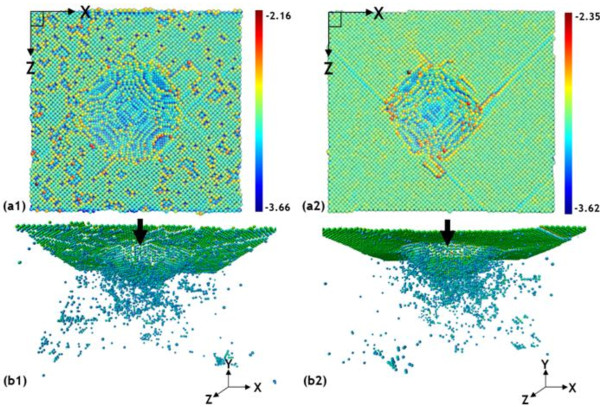
**Atomic potential energy views.** The atomic potential energy of the machining-induced surface and pristine single-crystal copper with two different perspective angles in the machining-induced surface and pristine single-crystal copper. (**a**_**1**_) and (**a**_**2**_), the top view on the machining surface; (**b**_**1**_) and (**b**_**2**_), the interior defects inside the specimen.

Figure 6 (a and b) shows atomic potential energy views of the specimen when the diamond indenter penetrates into the specimen with a depth of 1.5 nm. The arrow indicates the nanoindentation penetration direction. The machining-induced surface in Figure 6 (a) reveals randomly distributed colors of atomic potential energy, implying the local structure transition of a perfect crystalline structure. The defects on the machining-induced surface can be clearly identified by the atomic potential energy for the value of atomic potential energy is remarkable. However, their value of them is much higher than that in the pristine single-crystal copper, as shown in Figure 6 (a_2_). These high-energy instability structures on the machining-induced surface easily propagate the dislocation-related defects beneath the surface in the specimen. In contrast, the dislocation loops in the machined specimen colored with the atomic potential energy, as shown in Figure 6 (b_1_), are with lower potential energy than those in the pristine single-crystal copper specimen, as shown in Figure 6 (b_2_). These results provide evidence of the influence of nanocutting process on single-crystal FCC metals and consequently on the physical properties of the machining-induced surface. We can confirm that the physical properties of the machining-induced surface have altered largely.

#### The hardness and Young's modulus of the machining-induced surface

**Figure 7:**
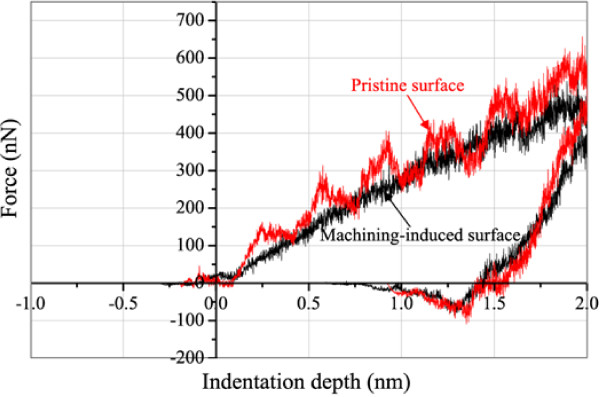
**Nanoindentation MD simulation load-displacement curves on the machining-induced surface and pristine single-crystal copper.** The indenter radius is 5.0 nm, and the maximum penetration depth is 2.5 nm.

The load and displacement data are monitored during the indentation process and then converted to the *P*-*h* curve which contains abundant information of the material, such as hardness, elastic modulus, and yield stress. Figure 7 is the load-displacement (or indentation depth) curve of a complete nanoindentation from the MD simulation. It mainly consists of two portions, loading and unloading processes.

In Figure 7, the loading curves of the two surfaces present some different characteristics. The discontinuity can be clearly observed as for the copper with perfect structure, which agrees with conventional studies. However, the loading curve of the machining-induced surface is much smooth. The differences are due to the dislocation nucleation-induced elastic and plastic deformation transformation. Compared to the maximum energy needed to be developed and propagated in the machining-induced surface, it is much larger in the pristine copper specimen. Since the high-energy initial defects have existed on the machining-induced surface, the power to trigger dislocation nucleation is less needed. When the dislocations emit from the dislocation nucleation and propagate in the specimen, the accumulated energy is released. Therefore, the amplitude value of the indentation curve on the pristine surface is much larger than that on the machining-induced surface.

According to the Oliver-Pharr method [6], nanoindentation hardness is defined as the indentation load divided by the projected contact area of the indentation. The indentation hardness (*H*) can be obtained at the peak load given by

(7) $$H=\frac{P_{\max}}{A_{c}}$$

where *P*_max_ is the peak load and *A*_*c*_ is the projected contact area. The projected contact area can be calculated from the relation as follows:

(8) $$A_{c}=\frac{\pi d_{c}{}^{2}}{4}$$

where *h*_*c*_ is the contact depth which is given by [20]

(9) $$h_{c}=h_{\max}-\epsilon \frac{P_{\max}}{S}$$

where *ϵ* is a constant and depends on the geometry of the indenter (*ϵ* = 0.72 for cone indenter, *ϵ* = 0.75 for paraboloid of revolution, and *ϵ* = 1.00 for flat indenter) [21]. *h*_max_ is the maximum penetration depth, and *S* is the contact stiffness. *A*_c_ is the projected contact area under the peak indentation depth.

The contact stiffness *S* can be calculated from the slope of the initial portion of the unloading curve and *S* = *dP*/*dh*, which can be obtained by curve fitting of 25% to 50% unloading data [22]. Based on relationships developed by Sneddon, the contact stiffness *S* can also be expressed by

(10) $$S=2\beta \sqrt{\frac{A}{\pi }}E_{r}$$

where *β* is a constant and depends on the geometry of the indenter (*β* = 1.034 for a Berkovich indenter, *β* = 1.012 for a Vickers indenter, and *β* = 1.000 for a cylinder indenter).

Because both the sample and the indenter have elastic deformation during the indentation process, the reduced modulus *E*_*r*_ is defined by

(11) $$\frac{1}{E_{r}}=\frac{1-v_{s}^{2}}{E_{s}}+\frac{1-v_{i}^{2}}{E_{i}}$$

where *E* and *ν* are the elastic modulus and Poisson's ratio for the sample; *E*_*i*_ and *ν*_*i*_ are the elastic modulus and Poisson's ratio for the indenter, respectively. For the diamond indenter, *E*_*i*_ = 1,141 GPa and *ν*_i_ = 0.07. The indenter was assumed to be rigid as mentioned above, and the value of *E*_*i*_ is infinite; *v*_*s*_ is equal to 0.278 [23].

According to the Oliver-Pharr method mentioned above, the nanoindentation hardness, contact stiffness, and elastic modulus of the materials can be obtained. The comparison of indentation depths at different loading stages are shown in Table 3.

**Table 3:** The applied load versus penetration depth in loading stage

		**Depth**			
		**0.5 nm**	**1.0 nm**	**1.5 nm**	**2.0 nm**
Applied load to the indenter (nN)	Machining-induced surface	118.83	246.22	336.51	522.40
	Pristine surface	167.74	268.15	487.05	530.47

Table 3 shows the comparison of indentation loads at different penetration depths of the pristine single-crystal copper specimen and machining-induced surface. It can be noted that the indentation loads on the machining-induced surface are much smaller than those on the pristine surface with the same indentation depth, respectively. No remarkable difference was found when the maximum indentation penetration depth is larger than 2.0 nm. The amplitude value of the indentation curve on the pristine surface is much larger than the other. It is due to the dislocation embryos which developed and propagated in the specimen under the diamond indenter. However, when the maximum penetration is smaller than 2.0 nm, the hardness of the diamond-turned surface becomes distinctly lower than that of the pristine copper. At a sufficiently small load, the indentation response will be mainly due to the surface effects. At a slightly larger indentation penetration depth, the indentation loads are much smaller than those of the pristine single-crystal copper surface. It can be concluded from these results that the machining-induced surface is softer than pristine single-crystal copper.

In conventional metal machining, the near-surface layer is much harder than the original material in the surface. Such a surface-hardening phenomenon is due to work-hardening effects. The work-hardening effects are often due to the dislocation winding near the crystal boundary, where the dislocations are not able to pass across the potential barrier. However, the surface-softening effect during machining is due to no crystal boundaries in single-crystal copper, and the dislocation activities are free to move.

It can also be noted that the calculated hardness of the pristine single-crystal copper specimen and machining-induced surface is 10.55 and 9.25 GPa by Equations 5, 6, 7, 8, 9, respectively, and the elastic modulus is 120.4 and 117.7 GPa, respectively. The machining-induced surface has a lower hardness than pristine single-crystal copper by about −12.3%, and the elastic modulus has no significant disparity (about 2.21%). The immobile dislocations on the machining-induced surface serve as the origin of mobile dislocations in the nanoindentation. The permanent plastic deformation is derived from the movement of dislocations. It has been revealed that the machining-induced surface would influence the physical properties of pristine single-crystal copper as well as other single-crystal FCC metals. The dislocations during nanocutting have been shown to play an important role in the formation of interior defects as well as surface profiles. Therefore, the accurate prediction of the thickness and mechanical properties of the machining-induced surface becomes vital when trying to use it in the application.

## Discussion

### The effect of cutting direction

Previous studies have introduced the concept of the subsurface damage layer after nanomachining. The criterion of the material damage nanocutting has a lot of statements, such as the thickness of the damage subsurface [3] and the variation of potential energy [2]. In fact, the dislocations distributed in the specimen alter the machining-induced surface mechanical properties. The immobile vacancy-related dislocations may lead to the nucleation of mobile dislocations.

**Figure 8:**
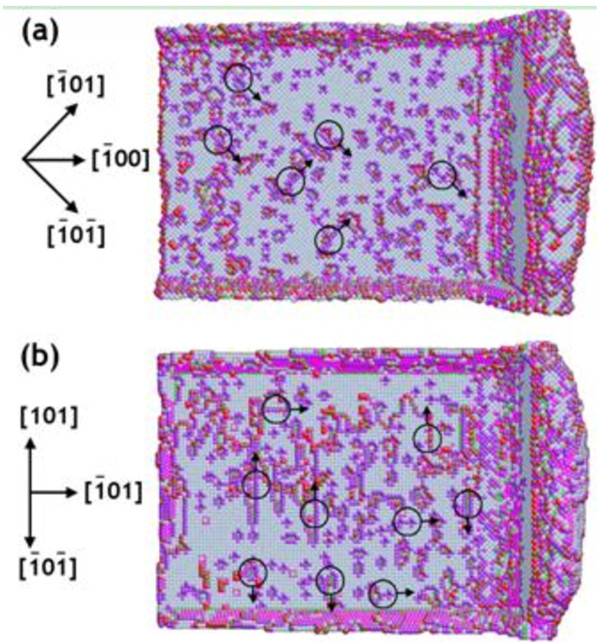
**The top view of the machining-induced surface after relaxation in two different cutting directions.** (**a**) Along [100] and (**b**) [101] directions.

Figure 8 shows the snapshots of the machining-induced surface after nanocutting in the [ī00] and [ī01] crystal directions on the (010) crystal surface, respectively. The distribution of immobile vacancy-related dislocations on the machined surface largely affects the properties of the machined surface. Since the immobile dislocations on the machining-induced surface lead to the nucleation of mobile dislocations, the quality and distribution of dislocations on the machine-induced surface determine the penetration of mobile dislocations in the specimen. When the cutting direction is along the [ī00] crystal orientation, most of the residual defects on the machining-induced surface prefer the [ī0ī] and [ī01] directions because they coincide with one of the three slip directions on this FCC (111) surface. Almost no defects are on other crystal orientations. The simulation is rather different on the other cutting direction, the [ī01] crystal orientation. As the nanocutting direction is along one of the three slip directions in the FCC crystal, most of the defects move along with the same direction with the cutting direction. Most of the residual defects on the machining-induced surface are making an angle of 90° with the cutting direction. In this case, most of the surface residual defects move to either [ī0ī] or [ī01] crystal orientations, which also run parallel with the three slip vectors in the FCC crystal. Because of the different cutting directions on the surface, the quality and distribution of residual defects in the damaged layer in the surface are not the same. Once the nanoindentation test begins, this balance is immediately broken, and the bulk glides are more likely to take place along specific directions. More details about the generated dislocations derived from the residual defects in the subsurface during nanoindentation are in the following paragraph.

**Figure 9:**
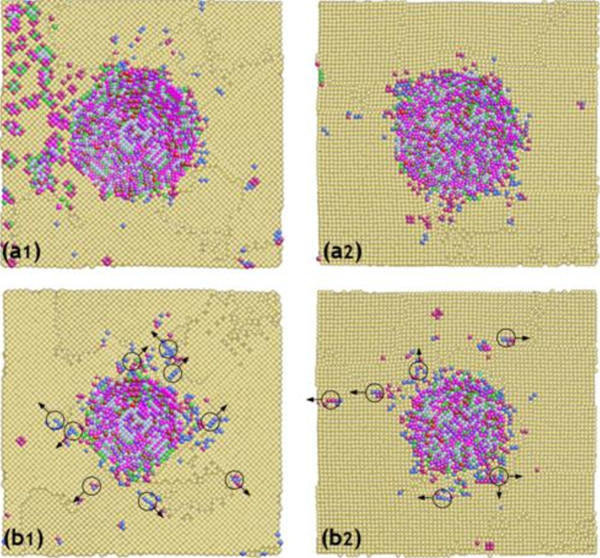
**Emission of dislocations.** The emission of dislocations in the subsurface during nanoindentation beneath the machining-induced surface along the [100] and [101] crystal orientations. (**a1**) and (**b1**), along the [100] cutting direction; (**a2**) and (**b2**), along the [101] cutting direction.

Figure 9 shows the emission of dislocations in the subsurface during nanoindentation beneath the machining-induced surface along the [ī00] and [ī01] crystal orientations, respectively. The machined layer on the surface is invisible for the immobile dislocations make it difficult to identify the newly generated dislocation loops in the surface due to nanoindentation. The movements of partial dislocation loops have often been found in nanoindentation simulations of single-crystal FCC metals in previous studies. They are of great importance in material deformation process because they mediate the plastic deformation. Figure 9 (a_1_ and a_2_) shows the cross-sectional view of the specimen beneath the machining-induced surface of 0.28 nm. More dissimilar glide patterns of surface dislocations around the diamond indenter are observed in Figure 9 (a_1_), which indicates that the extent of the damaged layer under the machined surface along [ī00] is larger than that along [ī01]. The defects around the indenter may lead to the nucleation of dislocations with large hydrostatic pressure under the diamond indenter. Figure 9 (b_1_ and b_2_) shows the cross-sectional view of the specimen beneath the machining-induced surface of 0.51 nm. The directions of the gliding dislocations in the subsurface are implied by the arrows attached to the small circles. The quantity and direction of the dislocations indicate that the subsurface damage is strongly dependent on the nanocutting directions. The number of the dislocations under the machining-induced surface along [ī00] is much larger than that along the [ī01] crystal orientation. As mentioned before, more dislocations beneath the indenter may lead to permanent plastic deformation easily. It is thus well inferred that the hardness of the machining-induced surface along the [ī00] direction is smaller than that along the [ī01] direction.

**Figure 10:**
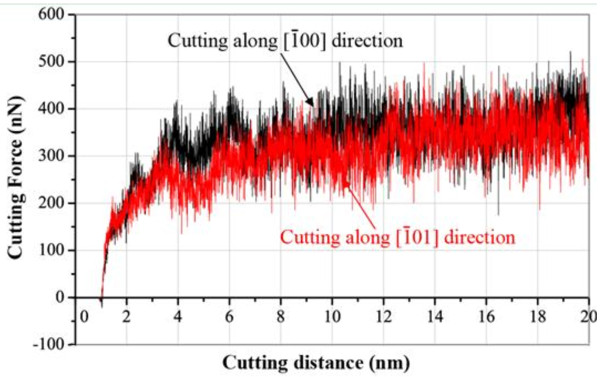
**Comparison of forces *F***_***x ***_**during the cutting processes along [ī00] and [ī01] crystal orientations, respectively.**

In order to have a clear understanding of the mechanism of the damaged layer after nanocutting, the cutting along two directions should be given. The interaction force, especially the *X*-direction load (*F*_*x*_) between the cutting tool and specimen, provides adequate pressure for nucleation and motion of dislocations which will lead to plastic deformation of the material in the specimen. In addition, the local pressure should be large enough for dislocations to pass through the other defects in the specimen. After the nanocutting process and a long enough stage of relaxation, the copper atoms on the machining-induced surface reconstruction and finally some vacancy-related defects are located on the surface, which derive from the propagation of dislocations in material deformation. The larger *F*_*x*_ results in a larger scale of glide directions in the specimen, which leads to much more serious plastic deformation underneath the tool. Figure 10 shows the variation of cutting force along the *X* direction on the specimen in the two models, respectively. Firstly, the cutting forces increase with the cutting tool thrust into the specimen. The curve is not smooth, and the value of pressure varies significantly. Then, the cutting forces are fluctuating around a certain value. It is obvious that the cutting force (*F*_*x*_) along the [ī00] direction is larger than that along the [ī01] direction. There are two reasons that may be responsible for this result. First, the process of dislocation nucleation under the cutting tool is continuous due to the cutting tool moving forward with high velocity; second, the motivation across dislocations underneath the cutting tool causes a great change in both the atomic structure and cutting force. For the same cutting parameters and crystal orientation along the *Y* direction, during the cutting process, the values of *F*_*y*_ are the same. More studies on how the dislocations influence the deformation along two cutting directions are stated in the following paragraph.

**Figure 11:**
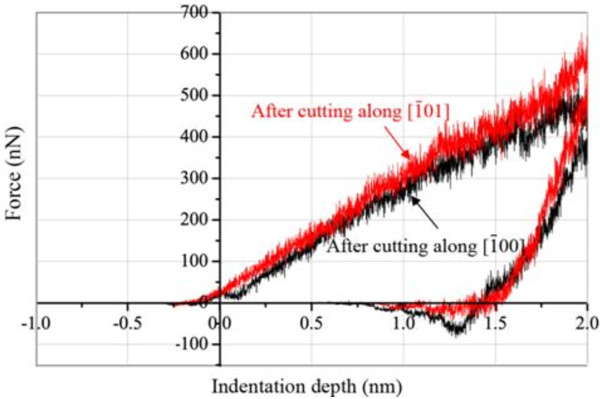
**Nanoindentation MD simulation load-displacement curves along different crystal directions, respectively.** Tip radius = 5.0 nm.

In order to measure the damage after nanocuttings along different crystal directions in quantity, the load-displacement (or indentation depth) curves of a complete nanoindentation from the MD simulation after nanocuttings are shown in Figure 11. It shows that at the maximum indentation depth of 2 nm, the indentation force is 540.89 nN along the cutting direction [ī00] and 651.70 nN along the cutting direction [ī01]. Table 4 compares the depths versus indentation depths in loading stage on the machining-induced surface along different cutting directions.

**Table 4:** The applied load versus penetration depth in loading stage

		**Depth**			
		**0.5 nm**	**1.0 nm**	**1.5 nm**	**2.0 nm**
Applied load to the indenter (nN)	Cutting direction [ī00]	118.83	250.14	406.03	522.40
	Cutting direction [ī01]	165.27	301.28	435.44	560.81

The variations of hardness and Young's modulus of the machining-induced surface with various cutting directions along different crystal orientations are calculated. The hardness of the machining-induced surface along [ī00] and [ī01] is 9.25 and 11.16 GPa by Equations 5, 6, 7, 8, 9, respectively, and the elastic modulus is 117.7 and 126.46 GPa, respectively. The machining-induced surface along [ī00] has lower hardness than the machining surface cutting along [ī01] by about −17.1%, and the elastic modulus has no significant disparity (about 6.9%). The comparison demonstrates that they are in excellent agreement with the anticipation that the cutting force along the different cutting directions on the same surface is not the same. Larger cutting force causes more severe damage in the subsurface, leading to more changes of the properties of the machined surface.

## Conclusion

The present investigation has shown how the machining-induced surface affects the mechanical properties in the atomic level of single-crystal copper by molecular dynamics simulation. Based on the above analysis, some interesting conclusions can be drawn as follows.

Hybrid potentials including the Morse and EAM potentials were employed to simulate the nanoindentation test on the machining-induced copper surface. The nanocutting simulation was carried out at the nanocutting velocity of 200 m/s. The simulation results show that some kinds of defects remain in the subsurface of the machining-induced surface. The defects in the damaged layer alter the mechanical properties of the machining-induced surface. When the indenter penetrated into the machining-induced surface after an adequate relaxation, the dislocation embryos derived from the vacancy-related defects are distributed in the subsurface. These results show that the hardness of the machined surface is smaller than that of single-crystal copper. In addition, the hardness and Young's modulus are calculated from the simulation results, which further verify the former analysis according to the motivation of dislocations in the specimen.

Then, the nanocutting was performed along different crystal orientations on the same crystal surface. It is shown that the crystal orientation directly influences the dislocation formation and distribution in the machining-induced surface. The crystal orientation of nanocutting is further verified to affect both dislocations and residual defect generations that are important in assessing the change of mechanical properties after nanocutting in this length scale.

## Endnote

^a^Distributed by Sandia National Laboratories, Albuquerque, NM, USA.

## Competing interests

The authors declare that they have no competing interests.

## Authors’ contributions

LZ conceived the research work, accomplished the framework of the manuscript, coordinated the collaboration, and participated in the simulation. HH did the proof reading of the manuscript. HZ did the literature review. ZM provided some basic inputs to the MD simulation and carried out the MD simulation. YY and XH helped revise the unsuitable grammar of the article. All authors read and approved the final manuscript.

## References

[B1] Fang FZ Wu H Zhou W Hu XT A study on mechanism of nano-cutting single crystal silicon J Mater Process Technol 2007 8 407 410 10.1016/j.jmatprotec.2006.12.007

[B2] Zhang JJ Sun T Yan YD Liang YC Dong S Molecular dynamics simulation of subsurface deformed layers in AFM-based nanometric cutting process Appl Surf Sci 2008 8 4774 4779 10.1016/j.apsusc.2008.01.096

[B3] Ikawa N Shimada S Tanaka H Ohmori G An atomistic analysis of nanometric chip removal as affected by tool–work interaction in diamond turning Ann CIRP 1991 8 551 554 10.1016/S0007-8506(07)62051-4

[B4] Ikawa N Shimada S Tanaka H Minimum thickness of cut in micromachining Nanotechnology 1992 8 6 9 10.1088/0957-4484/3/1/002

[B5] Shimada S Ikawa N Tanaka NH Ohmori G Uchikoshi J Feasibility study on ultimate accuracy in microcutting using molecular dynamics simulation Ann CIRP 1993 8 91 94 10.1016/S0007-8506(07)62399-3

[B6] Oliver WC Pharr GM An improved technique for determining hardness and elastic modulus using load and displacement sensing indentation experiments J Mater Res 1992 8 1564 1583 10.1557/JMR.1992.1564

[B7] Yan JW Takahashi H Tamaki J Gai XH Nanoindentation tests on diamond machined silicon wafers Appl Phys Lett 2005 8 181913 10.1063/1.1924895

[B8] Yan JW Takahashi H Tamaki J Gai XH Kuriyagawa T Transmission electron microscopic observation of nanoindentations made on ductile-machined silicon wafers Appl Phys Lett 2005 8 211901 10.1063/1.2133908

[B9] Zhao HW Shi CL Zhang P Zhang L Huang H Yan J Research on the effects of machining-induced subsurface damages on mono-crystalline silicon via molecular dynamics simulation Appl Surf Sci 2012 8 66 71

[B10] Cai MB Li XP Rahman M Study of the temperature and stress in nanoscale ductile mode cutting of silicon using molecular dynamics simulation J Mater Process Tech 2007 8 607 612

[B11] LAMMPS Molecular Dynamics Simulator 2011 http://lammps.sandia.gov/

[B12] Foiles SM Baskes MI Daw MS Embedded-atom-method functions for the fcc metals Cu, Ag, Au, Ni, Pd, Pt, and their alloys Phys Rev B 1986 8 7983 10.1103/PhysRevB.33.7983 9938188

[B13] Cai MB Li XP Rahman M Study of the mechanism of nanoscale ductile mode cutting of silicon using molecular dynamics simulation Int J Mach Tool Manuf 2007 8 75 80 10.1016/j.ijmachtools.2006.02.016

[B14] Cheong WCD Zhang LC Molecular dynamics simulation of phase transformation in silicon monocrystals due to nano-indentation Nanotechnology 2000 8 173 180 10.1088/0957-4484/11/3/307

[B15] Plimpton S Fast parallel algorithms for short-range molecular dynamics J Comput Phys 1995 8 1 19 10.1006/jcph.1995.1039

[B16] Ju L Van Vliet KJ Ting Z Sidney Y Subra S Atomistic mechanisms governing elastic limit and incipient plasticity in crystals Nature 2002 8 307 310 10.1038/nature00865 12124619

[B17] Jun S Lee Y Kim SY Im S Large-scale molecular dynamics simulations of Al(111) nanoscratching Nanotechnology 2004 8 1169 1174 10.1088/0957-4484/15/9/011

[B18] Sang HO Marc L Daniel K In situ observation of dislocation nucleation and escape in a submicrometre aluminium single crystal Nature Mater 2009 8 95 100 10.1038/nmat2370 19151703

[B19] Zhou X Zhu Z Lin J Evolution of workpiece microstructure and cutting force during ultraprecision vibration assisted machining J Comput Theor Nanos 2013 8 78 85 10.1166/jctn.2013.2661

[B20] Sneddon IN The relation between load and penetration in the axisymmetric Boussinesq problem for a punch of arbitrary profile Int J Eng Sci 1965 8 47 57 10.1016/0020-7225(65)90019-4

[B21] Fischer-Cripps AC Nanoindentation 2004 New York: Springer

[B22] Oliver WC Pharr GM Measurement of hardness and elastic modulus by instrumented indentation: advances in understanding and refinements to methodology J Mater Res 2004 8 3 20 10.1557/jmr.2004.19.1.3

[B23] Lu CJ Bogy DB The effect of tip radius on nano-indentation hardness tests Int J Solids Struct 1995 8 1759 1770 10.1016/0020-7683(94)00194-2

